# Strategies for accelerating osteogenesis through nanoparticle-based DNA/mitochondrial damage repair

**DOI:** 10.7150/thno.77089

**Published:** 2022-08-29

**Authors:** Hye Jin Kim, Hui Bang Cho, Sujin Lee, Jiyon Lyu, Hye-Ryoung Kim, Sujeong Lee, Ji-In Park, Keun-Hong Park

**Affiliations:** 1 Laboratory of Nano-regenerative Medicine, Department of Biomedical Science, College of Life Science, CHA University, CHA Biocomplex, Sampyeong-Dong, Bundang-gu, Seongnam-si, 13488, Republic of Korea; 2 School of Medicine, CHA University, CHA Biocomplex, Sampyeong-Dong, Bundang-gu, Seongnam-si, 13488, Republic of Korea

**Keywords:** PEI, RS-1, DNA damage, Mitochondrial stability, Osteogenesis

## Abstract

The efficiency of gene therapy is often dictated by the gene delivery system. Cationic polymers are essential elements of gene delivery systems. The relatively cheap cationic polymer, polyethyleneimine, has high gene delivery efficiency and is often used for gene delivery. However, the efficiency of gene therapy with polyethyleneimine-pDNA polyplex (PEI) is low. Human mesenchymal stem cells transfected with polyethyleneimine and a plasmid carrying the important osteogenic differentiation gene runt-related transcription factor 2 (RUNX2) accumulated DNA double-strand breaks and mitochondrial damage proportional to the amount of polyethyleneimine, reducing viability. Genomic/cellular stabilizer mediating RUNX2 delivery (GuaRD), a new reagent incorporating RS-1 NPs developed in this study, promoted DNA repair and prevented the accumulation of cell damage, allowing the delivery of pRUNX2 into hMSCs. while maintaining genome and mitochondrial stability. DNA damage was significantly lower and the expression of DNA repair-related genes significantly higher with GuaRD than with PEI. In addition, GuaRD improved mitochondrial stability, decreased the level of reactive oxygen species, and increased mitochondrial membrane potential. Osteogenic extracellular matrix (ECM) expression and calcification were higher with GuaRD than with PEI, suggesting improved osteogenic differentiation. These results indicate that lowering the cytotoxicity of PEI and improving cell stability are key to overcoming the limitations of conventional gene therapy, and that GuaRD can help resolve these limitations.

## Introduction

Gene transfection into cells is an established technique to induce cellular functions. Both viral and non-viral vectors have been used as carriers to safely deliver these genes; however, this can lead to cellular stress, which can interfere with the functions of transfected genes 1-4. Therefore, we must consider the reagents used and the intrinsic level of cellular stress, as well as the stress induced by the gene delivered, when conducting gene transfection experiments.

Polyethyleneimine is a well-known, non-viral vector for gene delivery during gene therapy. Polyethyleneimine has many cations that readily bind to anionic DNA, which becomes internalized before delivery into cells 5-9. However, polyethyleneimine has been shown to be cytotoxic. It has been reported that the polyethyleneimine-pDNA polyplex (hereafter referred to as PEI) induces genotoxicity in various cell types. For instance, a 25 kDa branched PEI induces DNA damage in A431 and Jurkat T cells 10-13. In addition, cytotoxicity occurs when multiple molecular weight PEI is used for the transfection of Neuro2A cells, and reactive oxygen species (ROS) and DNA damage are induced in proportion to the molecular weight of polyethyleneimine 14, 15. PEI has been widely reported to cause mitochondrial damage. Mitochondrial membrane potential (MMP) is lost following PEI treatment, leading to cytochrome C leakage into the cytoplasm, inducing apoptosis 16.

Mitochondrial damage slows the differentiation of stem cells. Studies have shown that mitochondrial dysfunction decreases osteogenic differentiation 17-19. In addition, mitochondrial DNA mass increases during osteogenic differentiation 20, 21. These studies indicate that mitochondria can regulate the differentiation of stem cells, stressing the importance of mitochondrial stability. There have been attempts to overcome these problems, but to no avail. Nanoparticles based on tocopherol, an antioxidant, were produced to suppress ROS generation and MMP loss 22-24, while acetylation of PEI was shown to suppress DNA damage and ROS accumulation 25.

DNA damage caused by ROS can induce inappropriate transcriptional activation, which can have deleterious effects on cells. Stem cells that accumulate DNA damage cannot perform some fundamental physiological activities, which reduces their differentiation efficiency. Therefore, it is essential to maintain cell stability.

Here, we show that RS-1 (3-(benzylsulfamoyl)-4-bromo-N-(4-bromophenyl)benzamide) suppresses DNA damage during PEI-mediated gene therapy. RS-1 has been widely reported as a stimulator of RAD51 recombinase (RAD51), a protein essential for homologous recombination (HR) during DNA repair 26-30. RS-1 treatment of mouse embryos during somatic cell nuclear transfer reprogramming improves genome stability and reprogramming efficiency 31. Also, RAD51 promotes the synthesis of mtDNA and maintains the integrity of the mitochondrial genome 32-35, which protects mitochondrial activity in mammalian oocytes 36. In addition, the inhibition of the expression of DNA repair proteins RAD51 and MRE inhibits mitochondrial function 37-39. Thus, DNA repair and mitochondrial stability are closely related to each other.

RUNX2 gene and PLGA NPs were used to induce osteogenic differentiation. RUNX2 is a well-known transcription factor involved in the osteogenic process. PLGA, a polymer, is suitable for bone differentiation because it has excellent biocompatibility and biodegradability and mechanical strength 40-42. In this study, the co-delivery of a plasmid expressing pRUNX2 with RS-1 NPs improved osteogenic differentiation via the stabilization of genomic and mitochondrial DNA, thereby reducing mitochondrial damage and increasing cellular stability. The efficiency of RUNX2-dependent osteogenesis was higher with a genomic/cellular stabilizer mediating RUNX2 delivery (GuaRD) than with PEI, suggesting that GuaRD could be used to develop new strategies for gene therapy.

## Experimental section

### Materials

Human MSCs were purchased from Lonza (Basel, Switzerland). DMEM high glucose, FBS, and DPBS were purchased from HyClone (Utah, USA). Polyethyleneimine (25k) and RS-1 (R9782) were purchased from Sigma Aldrich (St. Louis, USA). Comet assay kit was purchased from R&D systems (Minneapolis, USA). Mitotracker Green AM, TMRE, ROS (DCFDA) staining dyes, and Live/Dead cell staining kit were purchased from Invitrogen (California, USA). ImmPRESS Duet double staining polymer and DAB staining kit was purchased from Vector Laboratories (California, USA).

### Synthesis of RS-1 NPs

RS-1 NPs were synthesized using an oil-in-water solvent evaporation technique. 1 g poly(D,L-lactide-co-glycolic acid (PLGA) was dissolved in 17 mL dichloromethane, and 5 mg RS-1 was dissolved in 100 μL DMSO. The RS-1 solution was emulsified with PLGA, dropped into 50 mL of a 2 % (w/v) aqueous PVA (12-23 kDa) solution, and mechanically stirred for 5 h at 500 rpm. The RS-1 NPs were stored overnight in a refrigerator, isolated, and then dialyzed at 4 °C for 24 h. After lyophilization, the solution was dissolved in distilled water at a concentration of 5 mg/mL.

### Characterization of two gene carriers

To confirm the shape and distribution, GuaRD and PEI were applied on carbon tape, dried completely, coated with Pt, and observed through a scanning electron microscope (SEM, Hitachi, Tokyo, Japan). The size and surface charge were measured by dynamic light scattering (DLS, Zetasizer Nano ZS, Malvern Panalytical, Malvern, UK). In addition, Fourier transform infrared (FTIR; IRspirit, Shimadzu, Japan) analysis was performed to confirm RS-1 synthesis. The sample was lyophilized and analyzed in its solid state.

### Evaluation of genomic stability

GuaRD was prepared by coating 0.5 mg of RS-1 NPs with 5 μg polyethyleneimine and adding 2 μg of pRUNX2. PEI was prepared by combining 5 μg of polyethyleneimine with 2 μg of pRUNX2. 2.5 x 10^5^ hMSCs were treated and analyzed after 6 h. RS-1 was used at a final concentration of 20 μM in DMSO. Single-cell gel electrophoresis (SCGE; comet assay) was performed as follows: cells were harvested, seeded in low melting temperature agarose, and lysed. After alkaline electrophoresis, the agarose was stained with Midori green and the slides were dried and observed under a CLSM (Olympus FV3000, Tokyo, Japan). mRNA was extracted from the cells using Trizol and sequenced. mRNA sequencing was outsourced to eBiogen (Seoul, Korea). The fixed cells were stained with γH2A.X antibody (ab22551, Abcam), and DNA DSBs foci were observed under a CLSM. Western blotting was performed on hemolyzed cells to quantify protein expression.

### Evaluation of mitochondrial stability

ROS dye-stained cells were treated with RS-1, PEI, or GuaRD and photographed for 5 min and 6 h. Staining intensity was quantified using CellSens software (Olympus, Japan). All other analyses were performed after 6 h had elapsed. After staining with TMRE and Mitotracker Green AM, cells were observed by CLSM. Cell TEM (Hitachi, Tokyo, Japan) was performed by outsourcing the fixed cells to Eulji University (Seongnam-si, Korea). For western blotting, p-DRP1 antibody (dynamin-related protein 1, #3455s, Cell signaling technology) NRF1 antibody (nuclear respiratory factor1, #46743, Cell signaling technology) and TOMM20 antibody (translocase of the mitochondrial membrane complex subunit 20; sc-17764, Santa Cruz) were used.

### Evaluation of osteogenic differentiation

Cells were treated with RS-1, PEI, or GuaRD for 6 h, and then removed and analyzed by 3D cell culture in a round-bottom tube for 4 weeks. mRNA was extracted from the cells using Trizol, and cDNA was synthesized. Real-time PCR was performed using SYBR (Takara, Japan). For western blotting, BSP antibody (bone sialoprotein; MAB1061, Millipore, Burlington, USA), COL I antibody (collagen type I, MAB3391, Millipore), and OCN antibody (osteocalcin, sc-74495, Santa Cruz, USA) were used. Cells were fixed with 4% PFA for 2 h and dehydrated with 2.5% sucrose for 2 h, and cryosection (Leica, Wetzlar, Germany) was performed. The tissue sections obtained were subjected to IF, IHC, and Alizarin red S staining. Areas specifically stained with tissue were quantified with Photoshop CS6.

### Statistical analysis

Statistical analysis was performed using the student's t-test in the SigmaPlot software. *P < 0.05, **P < 0.01, and ***P < 0.001 were considered to be statistically significant.

## Results and Discussion

### Characterization of GuaRD

**Figure 1:**
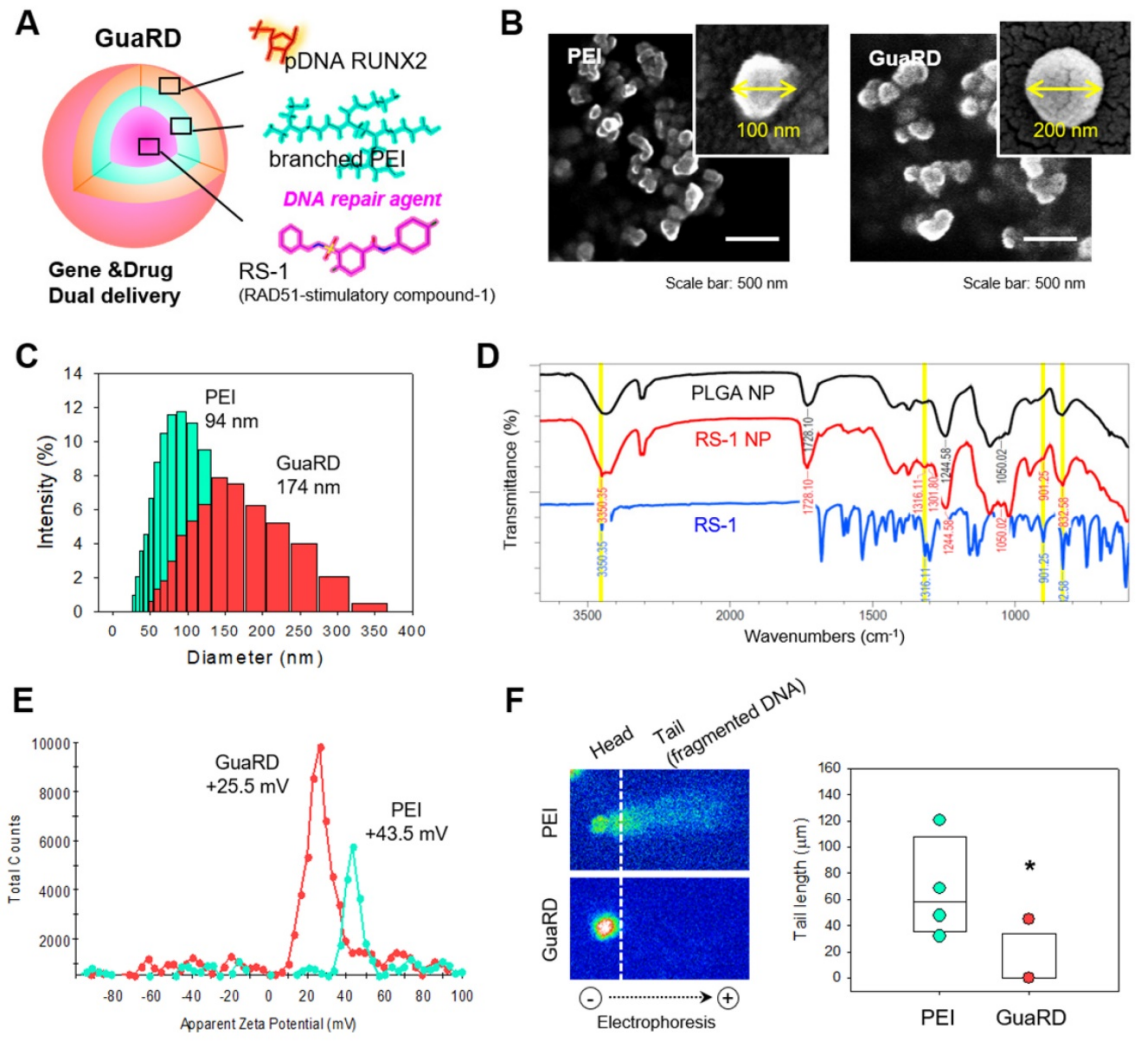
** Comparison of the characteristics of GuaRD and PEI.** (A) Diagram of GuaRD composition. pRUNX2 was loaded onto the surface of PEI-coated PLGA NP loaded with RS-1. (B) Distribution and shape of PEI and GuaRD analyzed by SEM. (C) Size distribution of PEI and GuaRD analyzed by DLS. (D) FTIR graph to examine whether RS-1 was loaded. Yellow highlighted peaks indicate RS-1-specific peaks. (E) Zeta potential of PEI and GuaRD analyzed by DLS**.** (F) Qualitative (right) and quantitative (left) analysis of genome stability using the SCGE assay.

RS-1 is a stimulator of the DNA repair factor RAD51. We loaded RS-1 into poly(lactic-co-glycolic acid) (PLGA) nanoparticles with the RUNX2 gene residing on the outside (**Figure 1A**). This complex was named genomic/cellular stabilizer mediating RUNX2 delivery (GuaRD; RS-1 NP-PEI-pRUNX2), while a simple polyplex formed between PEI and plasmid DNA was denoted as PEI. The structures of PEI and GuaRD were observed by SEM (**Figure 1B-C**). GuaRD had a spherical shape with an average size of 174 nm and a zeta potential of 25.5 mV, while PEI formed a more densely spherical particle of a smaller average size (94 nm) and zeta potential of 43.5 mM (**Figure 1C and E**). These data confirmed that both gene carriers had surface charge and were of sufficient size to allow cell entry.

RS-1 NP was confirmed by FTIR using PLGA NP and RS-1 as controls (**Figure 1D**). Peaks appeared in both the RS-1 NP and RS-1 sample at similar positions: 3350.35, 1361.1, 901, 832.58 cm^-1^ (yellow highlighted peaks); however, these peaks were not present in the PLGA NP sample. This suggests that RS-1 was efficiently loaded into the PLGA NP.

Next, we transfected RUNX2 into hMSCs by PEI or GuaRD, and performed SCGE assay to examine genome stability (**Figure 1F**). When PEI was used as a carrier, DNA fragmentation occurred; however, this was not the case with GuaRD, as evidenced by the far shorter DNA tail. These data suggest that GuaRD induces far less cell damage and genome instability than PEI.

### Cellular stress and cytotoxicity induced by PEI and GuaRD

**Figure 2:**
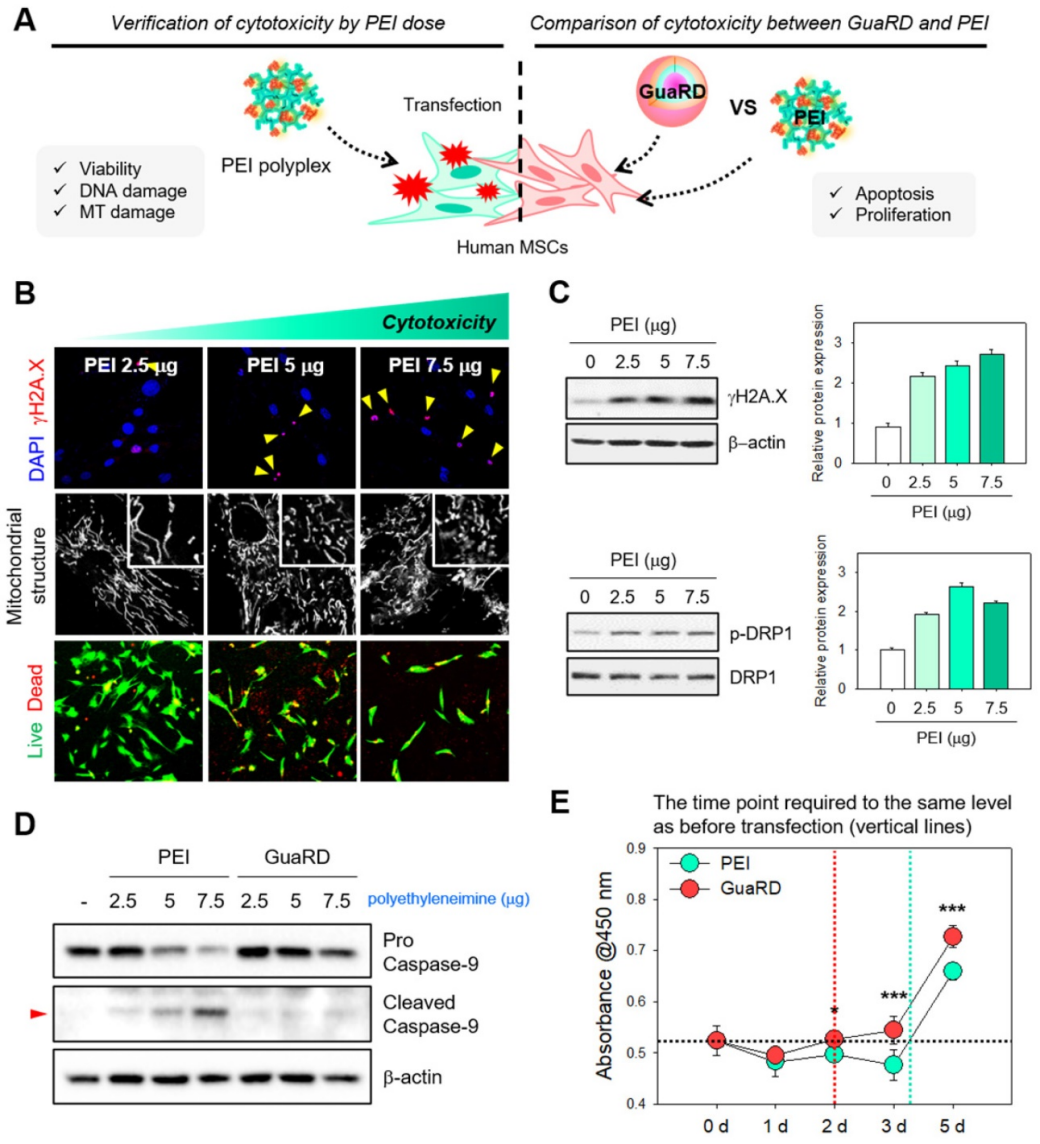
** Evaluation of cellular stress and cytotoxicity induced by PEI and GuaRD.** (A) Schematic diagram of the experiments conducted. (B) Evaluation of cellular stress induced by polyethyleneimine. DSB foci (red), mitochondrial structure, and live (green)/dead (red) cell staining are shown in the top, middle, and bottom panels, respectively. (C) DNA damage (top panel) and mitochondrial stress (bottom panel) induced by PEI. (D) Caspase 9 cleavage used as a marker of apoptosis induced by PEI and GuaRD. (E) Comparison of cell proliferation in GuaRD- and PEI-treated cells over 5 days. The dotted vertical lines indicate the time at which GuaRD (red) and PEI (mint) proliferation recovered to the same level as on day 0 (*p-value < 0.05, ***p-value < 0.001).

We next wanted to compare the effects of GuaRD and PEI on cells using several different assays (**Figure 2A**). Cytotoxicity, DNA damage, and mitochondrial dysfunction were observed when cells were treated with PEI in a dose-dependent manner (**Figure 2B**). As the PEI dose increased, γH2A.X foci, a marker for DNA double-strand breaks (DSBs), similarly increased, and nuclear condensation was observed (**Figure 2B** top panel, red). Similarly, proportional fragmentation of the mitochondrial structure occurred with increasing PEI (**Figure 2B** middle panel). These results were confirmed by western blotting for γH2A.X and the mitochondrial fission marker p-DRP1 (**Figure 2C**). DNA DSBs and mitochondria fission occur due to cell damage, and if the damage is not repaired and accumulates, it eventually leads to apoptosis 43-46. As the amount of PEI increased, the expression levels of γH2A.X and p-DRP1 increased. This result suggests that there was a correlation between cell damage and the amount of PEI, further suggesting that gene delivery using PEI is detrimental to cells.

This damage accumulation correlated with a decrease in cell viability, estimated using Live/Dead staining (**Figure 2B** bottom panel). The cytotoxicities of PEI and PLGA NP treatments were reduced by co-treatment with RS-1 NPs (**Figure S1**). Finally, we selected an optimal dose of GuaRD by examining the accumulation of γH2AX and p-DRP1 by western blotting (**Figure S2**). The lowest dose needed to suppress γH2AX and p-DRP1 accumulation was 400 μg; thus, this dose was used in the remainder of the study.

We wanted to determine whether the difference in cytotoxicity between PEI and GuaRD is driven by differences in the level of apoptosis. Western blotting for caspase 9 was performed to compare the cell toxicities of the PEI and GuaRD carriers. Caspase 9 cleavage, a marker for early apoptosis, was observed with 5 mg PEI but not with 5 mg GuaRD (**Figure 2D and S3**). Irreparable cell damage was induced by 7.5 mg PEI; therefore, 5 mg of each carrier was used to measure transfection efficiency (**Figure S4**). Transfection efficiency was 15.4% for PEI and 15.8% for GuaRD. A comparison of the transfection efficiencies of MSCs with PEI and lipofectamine showed that PEI is slightly more effective 47, 48. Similarly, PGA NPs resulted in higher transfection efficiencies than lipofectamine of Jurkat, SH-SY5Y and HeLa cells 49. PEI has higher transfection efficiency than another gene carrier, the PAMAM dendrimer 50. We used PEI as a positive control because PEI and nanoparticles are the most effective carriers.

Finally, cell proliferation was evaluated using the WST-1 assay (**Figure 2E**). Recovery from damage occurred 2 days after gene delivery; however, cells treated with GuaRD proliferated significantly faster than those treated with PEI, suggesting better recovery.

### Genome instability following PEI and GuaRD treatment

**Figure 3:**
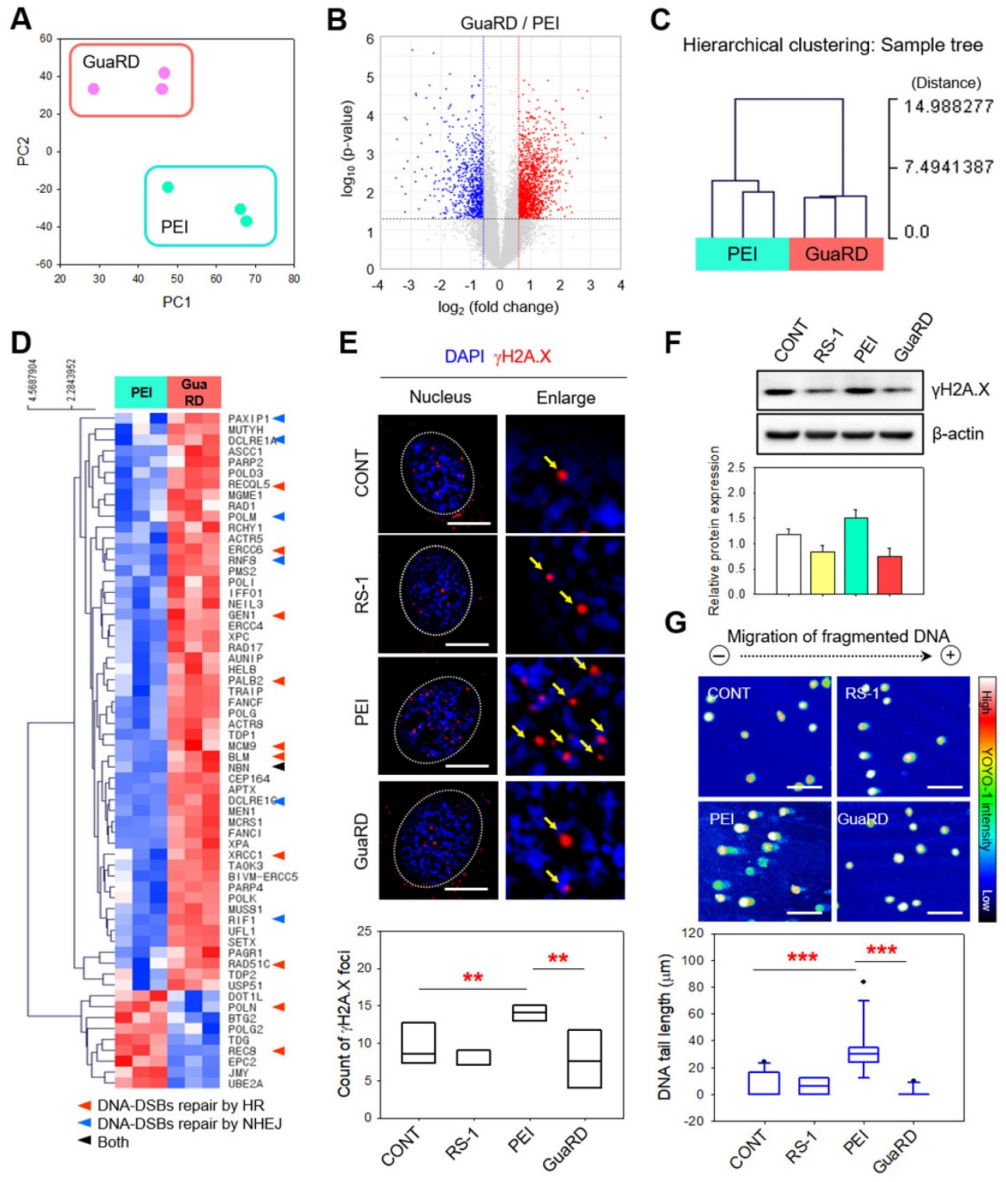
** Comparison of genome stability and transcriptome expression following GuaRD and PEI treatment. Significant differences were considered those with fold changes >1.5 and a p-value <0.05.** (A) PC plot showing the level of transcriptome similarity following PEI and GuaRD treatment. Three PEI- (mint) and GuaRD-treated (pink) samples were used. (B) Volcano plot of the differences in the transcription of individual genes induced by GuaRD and PEI. Increased and decreased expression are indicated in red and blue, respectively. Hierarchical clustering heat map (D) and sample tree (C) of the experiment in (B). Immunofluorescence staining (E) and western blotting (F) for γH2A.X. Scale bar, 10 μm. (G) SCGE assay and quantification. Scale bar, 150 μm.

Next, we used cluster analysis to examine gene expression patterns in hMSCs following PEI (green) or GuaRD (pink) treatment (**Figure 3A**). We found very little similarity between the gene expression patterns induced by these two carriers. We found increases (red) and decreases (blue) in gene expression following GuaRD treatment relative to the gene expression following PEI treatment (**Figure 3B**). We next performed gene ontology analysis to determine whether the genes associated with cell damage were differentially expressed between treatments (**Figure S5**). Sixty-two DNA repair genes with significant differences in expression were identified (**Figure 3C**). Three PEI samples formed clusters with a distance of 7.45 or less, while three GuaRD samples also formed clusters. Since the group forming the cluster means that the expression patterns are highly similar, the results of our independent experiments were highly consistent. On the other hand, the distance between the PEI and GuaRD clusters was around 15, suggesting that the expression patterns of the two treatments were very different. Expression of genes associated with DSB repair were more upregulated by GuaRD treatment than by PEI treatment (**Figure 3D**; red indicates upregulation, blue indicates downregulation). Genes associated with HR, non-homologous end joining (NHEJ), or both were among those upregulated (red, blue, and black arrows, respectively, in** Figure 3D**; **Figure S6**), suggesting that GuaRD promotes the repair of DSBs induced by transfection via the HR and NHEJ pathways.

Moreover, the expressions of 49 cell-cycle-associated genes were differentially expressed following PEI or GuaRD treatment (**Figure S7**). Twenty of these genes were associated with both DNA repair and the cell-cycle, of which 19 were more highly expressed by GuaRD treatment than by PEI treatment (**Figure S8**).

Next, we examined γH2A.X foci formation, a surrogate for DSB formation, in the nucleus of hMSCs cells treated for 6 h with vehicle (CONT), RS-1, PEI, or GuaRD (**Figure 3E**). PEI treatment induced many discrete γH2A.X foci (red dot, yellow arrow), while RS-1 and GuaRD had little effect. In addition, the level of γH2A.X protein detected by western blotting was lower following RS-1 treatment than in the vehicle control, and lower in GuaRD-treated cells than in PEI-treated cells (**Figure 3F**). This was further confirmed by SCGE (**Figure 3G**). PLGA NP alone similarly induced γH2A.X accumulation; however, this was lost upon co-treatment with RS-1 (**Figure S9**). Indeed, RS-1 co-treatment led to a reduction in γH2A.X in PEI-treated cells. Together, these data suggest that PEI triggers DNA fragmentation that is suppressed by RI-1 NPs.

### Evaluation of mitochondrial stability by transcriptome analysis

**Figure 4:**
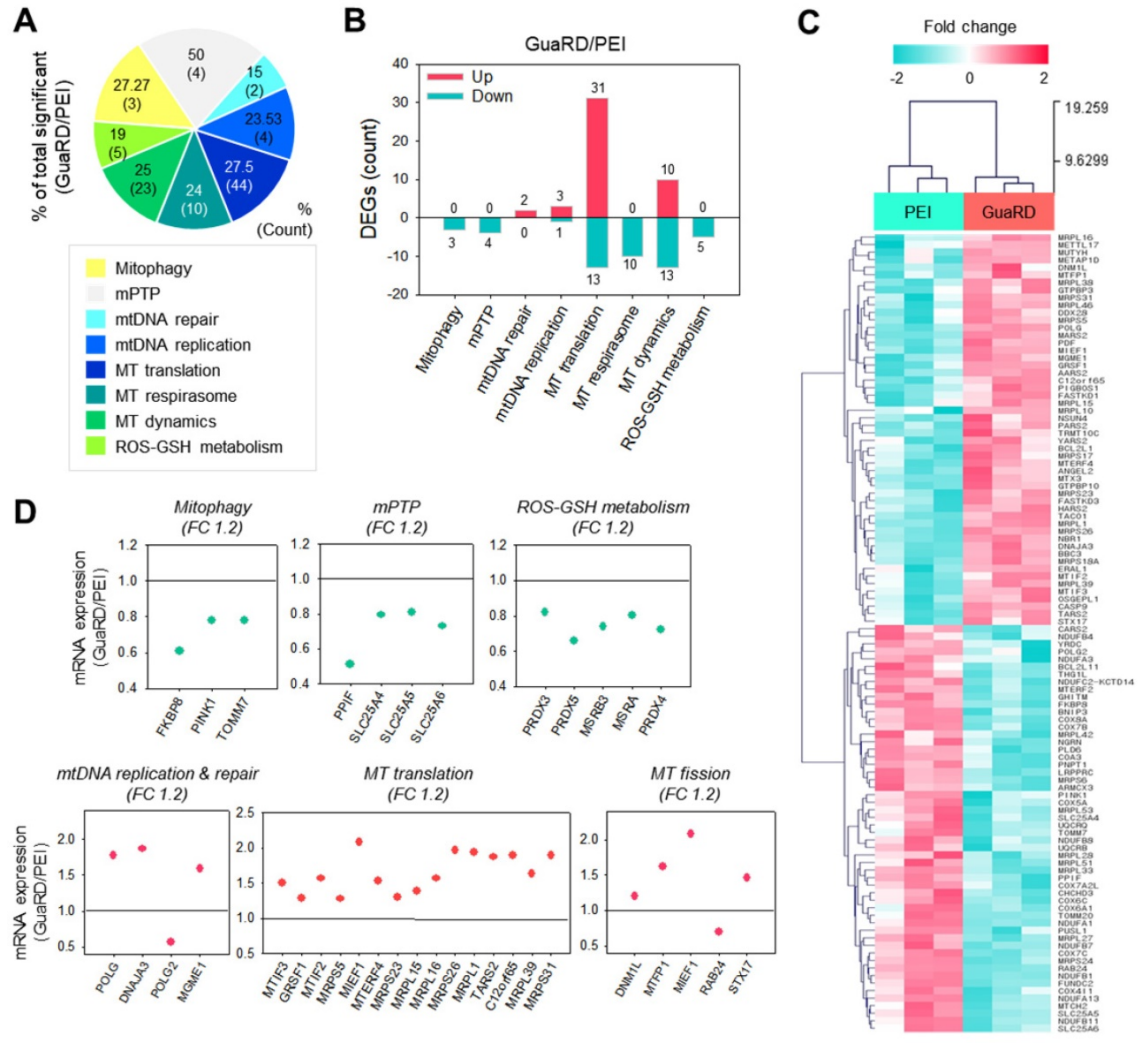
** Prediction of the effect of GuaRD on mitochondrial stability using transcriptomics.** (A-B) Changes in gene expression according to mitochondria-related GO term. Increased and decreased gene expressions are indicated in pink and mint, respectively (B). (C) The hierarchical clustering gene expression of genes identified in A and B. (D) Relative mRNA expression of representative genes by GO category. The green and pink scatterplots show increases (pink) and decreases (mint) in expression, respectively, following GuaRD treatment with respect to gene expression in PEI-treated cells. Significant differences were considered those a fold change >1.2 and a p-value <0.05.

Next, we performed transcriptome analysis to examine mitochondrial stability. Mitochondria-related gene ontology (GO) terms were classified into eight categories (Mitophagy, Mitochondrial permeability transition pore (mPTP), mtDNA repair, mtDNA replication, Mitochondrial (MT) translation, MT respirasome, MT dynamics, ROS-GSH metabolism), and genes with significant differences in expression between PEI and GuaRD treatment were identified (**Figure 4A**). **Figure 4B** shows the number of genes with increases (pink) or decreases (mint) in expression following GuaRD treatment relative to the gene expression in PEI-treated cells. The expression of genes involved in mtDNA repair, mtDNA replication, MT translation, and MT dynamic category was higher in GuaRD-treated cells than in PEI-treated cells. This suggests that GuaRD may induce mtDNA repair and activate mitochondrial biogenesis.

Fifty-two genes with significant differences in expression following GuaRD or PEI treatment were identified (**Figure 4C**). These genes were examined further by GO (**Figure 4D**), which revealed that the expression of genes related to mitophagy, mPTP, and ROS-GSH metabolism, the expression of which has been shown to increase following mitochondrial damage induced by external environmental changes, was decreased in GuaRD-treated cells (green scatter plot). Conversely, we found that the expression of genes belonging to mtDNA replication and repair, MT translation, and MT fission GO increased (pink scatter plot). This suggests that even if mitochondria are damaged by the PEI contained within GuaRD, they are actively regenerated inside the cell due to the release of RS-1 NPs. In addition to these results, previous studies have reported that the expression of DNA repair-related proteins is linked to the oxygen consumption rate (OCR). For instance, OCR is decreased when the protein expression of MRE and RAD51 is suppressed.^37^-^39^ We confirmed that the expression of RAD51 was increased by GuaRD, which presumably increased the OCR. Overall, our results show that improved genomic stability leads to an increase in mitochondrial stability.

### The effect of GuaRD treatment on mitochondrial stability

**Figure 5:**
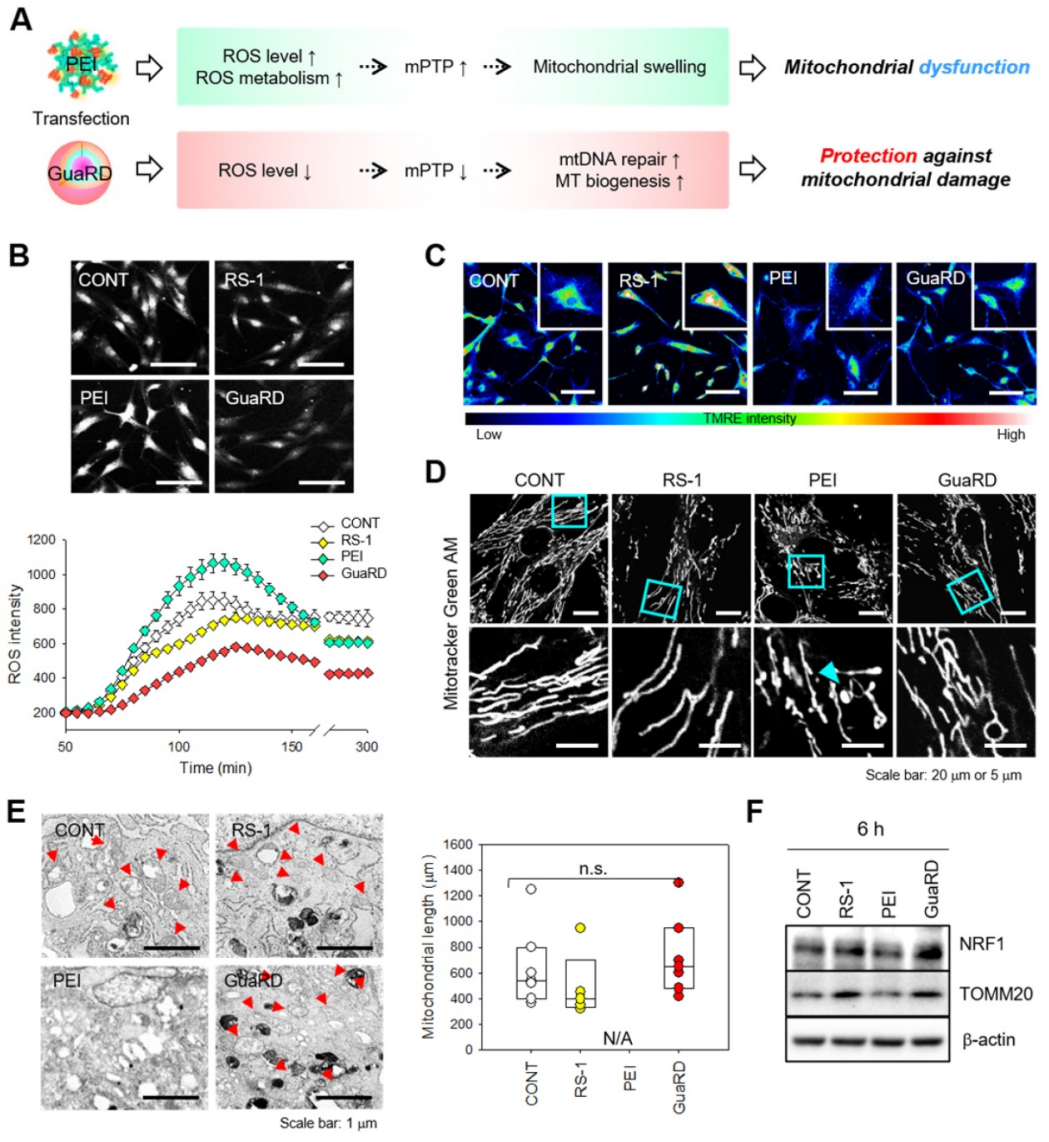
** The effect of GuaRD on mitochondrial stability.** (A) Predicted effects of the two gene carriers on mitochondria. (B) ROS levels 6 hours post-treatment. Scale bar, 200 μm. (C) Confirmation of MMP levels after 6 hours of treatment by TMRE staining. Scale bar, 80 μm. (D) Evaluation of mitochondrial structure and length after 6 hours of treatment by CLSM (D) and TEM (E). Cyan arrows indicate swollen mitochondria, and red arrows indicate normal mitochondria. (F) Analysis of mitochondrial biogenesis by western blotting.

We predicted that MSCs transfected with PEI would have 1) increased expression of metabolism-related genes associated with increased ROS, 2) decreased MMP due to an increase in mPTP, and 3) increased mitophagy to degrade damaged mitochondria. Conversely, in MSCs transfected with GuaRD, we predicted that repair or replication of damaged mtDNA and MT replication and fission would increase (**Figure 5A**). To test this hypothesis, hMSCs were treated with vehicle (CONT), RS-1, PEI, or GuaRD, and ROS levels were evaluated over time for 6 h (**Figure 5B**). Treatment with RS-1 led to lower ROS production than the control treatment. Similarly, GuaRD treatment strongly reduced ROS production, while ROS production following PEI treatment was greater than after the control treatment. These data suggest that RS-1 prevents the rapid generation of ROS.

TMRE staining was performed to examine the MMP level (**Figure 5C**). The higher the TMRE fluorescence intensity, the greater the MMP. The highest intensity was observed in RS-1-treated cells, while PEI treatment greatly decreased TMRE intensity. Cells treated with GuaRD had a higher TMRE intensity than those treated with PEI. These data suggest that PEI causes mitochondrial stress and substantially reduces MMP, which can be reversed by RS-1 NPs.

We further observed mitochondrial distribution and structure using Mitotracker staining (**Figure 5D**). Following PEI treatment, mitochondrial swelling and shortening were observed due to extreme mitochondrial damage. However, this was not the case in GuaRD-treated cells, suggesting that RS-1 maintains a stable mitochondrial structure. These data were confirmed using Cell TEM (**Figure 5E and S10**). RS-1 and GuaRD-treated cells exhibited crista, a structural feature of mitochondria, with lengths similar to those of the control. However, no normal mitochondria were observed in PEI-treated cells.

Following PEI treatment, ROS increased, mitochondria were damaged, and mitochondrial division was observed. However, in GuaRD-treated cells, mitochondria did not divide and were stable. This mitochondrial stability improved cell proliferation and differentiation. Finally, the expression of mitochondrial biogenesis markers was examined by western blotting (**Figure 5F and S11**). The expression of NRF1, an MT biogenesis marker, and TOMM20, an MT outer membrane marker, was higher in RS-1 NP- and GuaRD-treated cells than in those treated with PEI, indicating that RS-1 promotes MT biogenesis, resulting in an increase in mitochondrial mass.

It is known that there is a close correlation between the differentiation of mitochondria and stem cells. Active mitochondria promote osteogenic differentiation and their mass increases during osteogenic differentiation.^17^-^20^ For this reason, our observation that the increase in mitochondrial stability following GuaRD treatment promotes osteogenic differentiation is important.

### Osteogenic differentiation following PEI or GuaRD treatment

**Figure 6:**
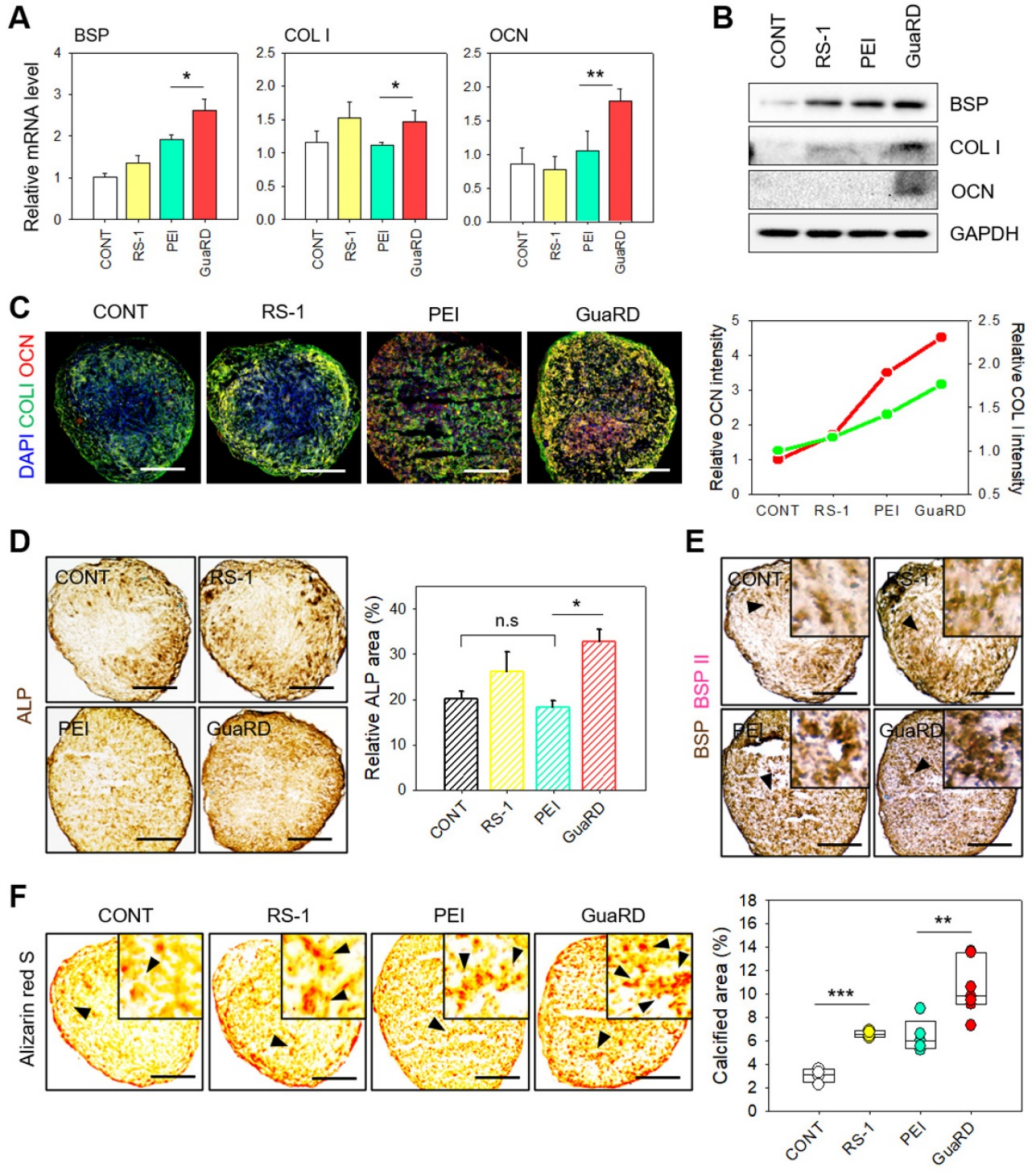
** The effect of GuaRD on osteogenic activation.** (A) Evaluation of osteogenic markers by analysis of mRNA expression (A) and protein level (B) (*p-value < 0.05, **p-value < 0.01). (C) Qualitative and quantitative evaluation of COLI and OCN expression by immunofluorescent staining. Scale bar, 400 μm. (D) Immunohistochemistry of ALP expression (*p-value < 0.05). (E) Dual immunohistochemistry of BSP I and II expression. (F) Qualitative and quantitative evaluation of calcification by Alizarin red S staining. Scale bar, 400 μm.

After evaluating the effects of PEI and GuaRD on genome and mitochondrial stability, we examined the effects of these carriers on bone cell differentiation after the delivery of RUNX2. We first examined the expression of the osteogenic markers BSP, COL I, and OCN by qRT-PCR and western blotting 4 weeks post-treatment (**Figure 6A-B and S12**). BSP and COL I expression was higher after RS-1 treatment than after the control treatment. RS-1 improved mitochondrial stability and activity, and, consequently, increased the expression of osteogenic ECM markers. PEI increased the expression of BSP induced by RUNX2, but not that of other ECM markers. Conversely, GuaRD treatment increased the expression of all ECM markers due to thesynergistic action of RS-1 and RUNX2. The osteogenesis effect of GuaRD was compared with that of PLGA NP and PLGA NP plus RS-1. ECM expression was, again, higher in GuaRD-treated cells. PLGA is a polymer with higher biocompatibility and osteoconductivity than other polymers used for bone regeneration. It has better biodegradability than HA and TCP and better mechanical strength than collagen and PCL, which makes it very suitable for bone regeneration.^40^-^42^ The higher expression of osteogenic ECM markers by PLGA NPs than by PEI may be due to the osteoconductivity of PLGA. Interestingly, PLGA NP plus RS-1 induced lower expression of ECM markers than PLGA NP alone. It is possible that treatment with the transfection reagent and drug together can lead to stem cell damage, resulting in decreased differentiation efficiency (**Figure S13**).

We further confirmed our results by examining the expression level of COL I (green) and OCN (red) by immunofluorescent staining and immunohistochemistry (IHC), which revealed that the most effective differentiation occurred in GuaRD-treated cells (**Figure 6C-D**). We examined the expression of osteogenic ECM markers BSPI (brown) and BSPII (pink) by IHC (**Figure 6E**). High levels of BSPI and BSPII expression were observed following GuaRD treatment, which was confirmed by OCN staining (**Figure S14**). These results suggest that GuaRD transfection into stem cells facilitates their differentiation into osteocytes.

Various factors promote the differentiation of stem cells into osteocytes. The secretion of minerals is an important measure of osteogenic differentiation since the ECM plays an important role in bone formation and remodeling, the regulation of calcium deposition, and mineralization. As a result, the formation of the ECM, such as calcium deposition, is evidence that stem cells have differentiated into osteocytes. Therefore, Alizarin red S staining was performed to evaluate the degree of calcification (Figure 6F). The results showed many calcium deposits in GuaRD-treated cells, suggesting that efficient differentiation of the bone cells had occurred.

## Conclusion

Although gene delivery is required to induce stem cell differentiation, cell damage can occur during this process. In this study, we found that mitochondrial dysfunction and DNA damage occurred after gene delivery using PEI. To address this problem, we produced a new gene delivery agent, GuaRD, containing RS-1 NPs, a substance that stimulates the DNA repair protein RAD51. Delivery of pRUNX2 using GuaRD into hMSCs significantly reduced DNA damage, prevented or inhibited mitochondrial function damage, and increased osteogenic differentiation. We also found that RS-1 induced bone differentiation. These results reveal that reducing genomic and mitochondrial stability is important for efficient stem cell differentiation. The results of our study open new perspectives for the promotion of stem cell differentiation and the development of new gene therapies.

## Supplementary Material

Supplementary figures.

## Figures and Tables

**Scheme 1:**
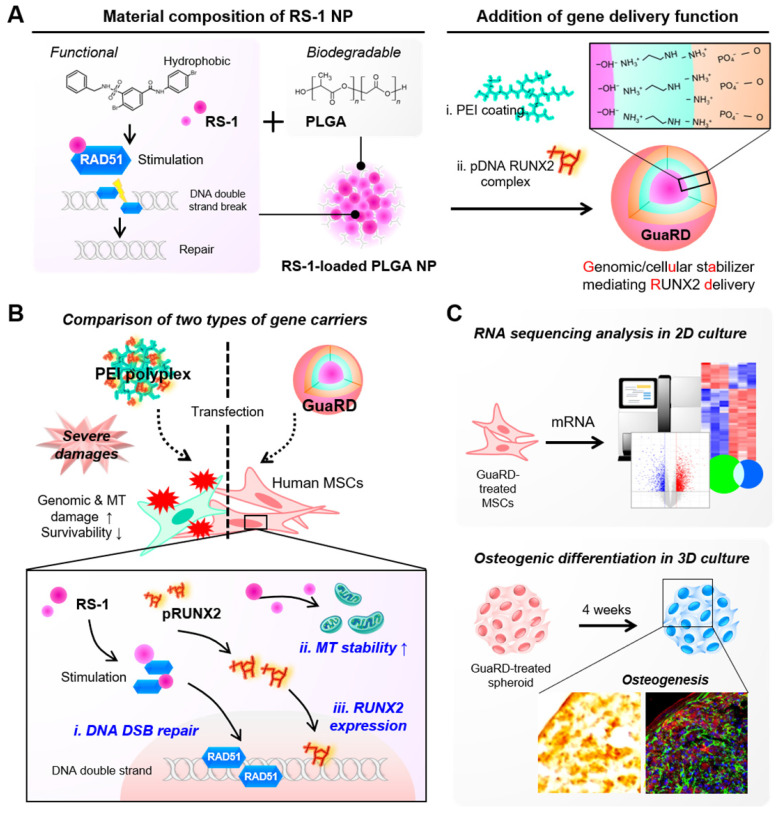
** Schematic diagram of the GuaRD configuration and its functions.** (A) The components and the process used to produce GuaRD. (B) The effects of PEI-pDNA polyplex (PEI) and GuaRD on cells. PEI causes genomic and mitochondrial damage, but GuaRD prevents this from happening. (C) The methods used to analyze the function of GuaRD.

## Abbreviations

RAD51: RAD51 recombinase
RUNX2: runt-related transcription factor 2
ECM: extracellular matrix
NPs: nanoparticles
PLGA: poly(D,L-lactide-co-glycolic acid
GuaRD: genomic/cellular stabilizer mediating RUNX2 delivery
PEI: pDNA-polyethyleneimine polyplex
DNA DSBs: DNA double-strand breaks
GO: gene ontology
MMP: mitochondrial membrane potential
mtDNA: mitochondrial DNA
mPTP: mitochondrial permeability transition pore
DRP1: dynamin-related protein 1
NRF1: nuclear respiratory factor 1
TOMM20: translocase of the mitochondrial membrane complex subunit 20
BSP: bone sialoprotein
COL I: collagen type I
OCN: osteocalcin
DLS: dynamic light scattering
SEM: scanning electron microscope
FTIR: Fourier transform infrared
