# Supplementary material for: Strategies for accelerating osteogenesis through nanoparticle-based DNA/mitochondrial damage repair
Source: Theranostics. 2022 Aug 29;12(14):6409–21. doi: 10.7150/thno.77089 (PMC9475457; doi:10.7150/thno.77089)
Supplement: Supplementary file 1 — Supplementary figures. [file thnov12p6409s1.pdf]

Supporting information

Strategies for Accelerating Osteogenesis through Nanoparticle-based DNA/Mitochondrial Damage Repair

Hye Jin Kim[1], Hui Bang Cho[1], Sujin Lee[1], Jiyon Lyu[2], Hye-Ryoung Kim[1], Sujeong Lee[1], Ji-In Park[1], and Keun-Hong Park[1]*

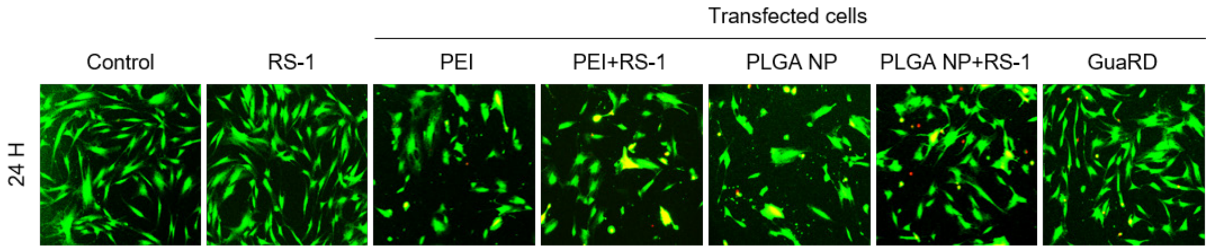

Figure S1. Evaluation of cytotoxicity by Live/Dead staining.

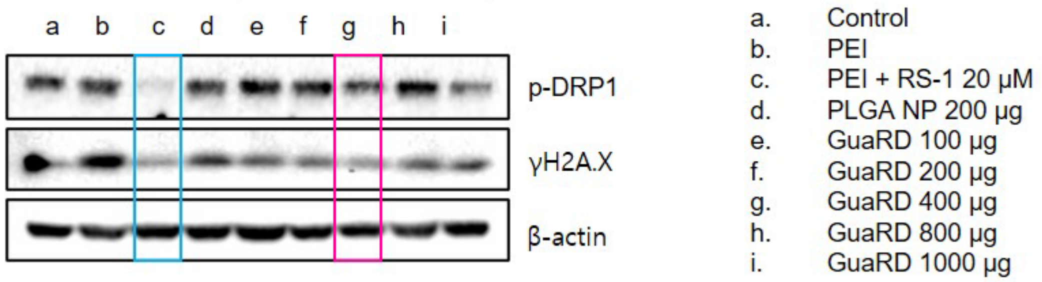

Figure S2. Evaluation of genomic and mitochondrial damage by western blotting.

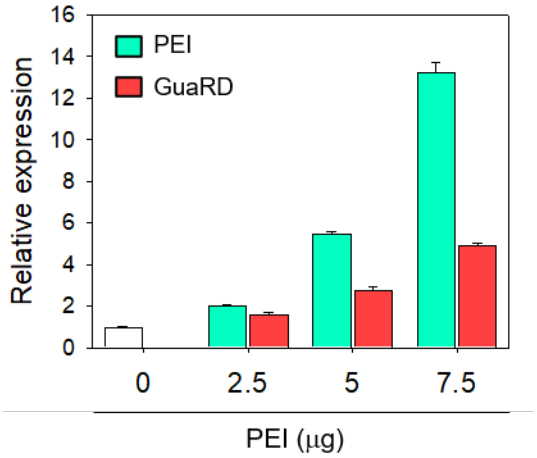

Figure S3. Quantitative graph for Figure 2D.

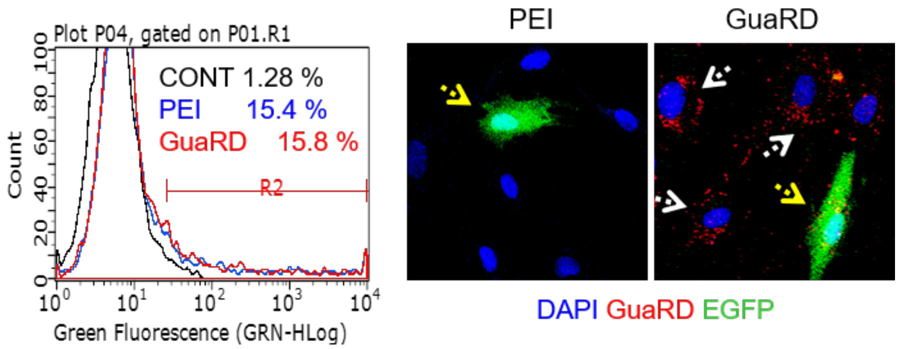

Figure S4. Confirmation of transfection efficiency.

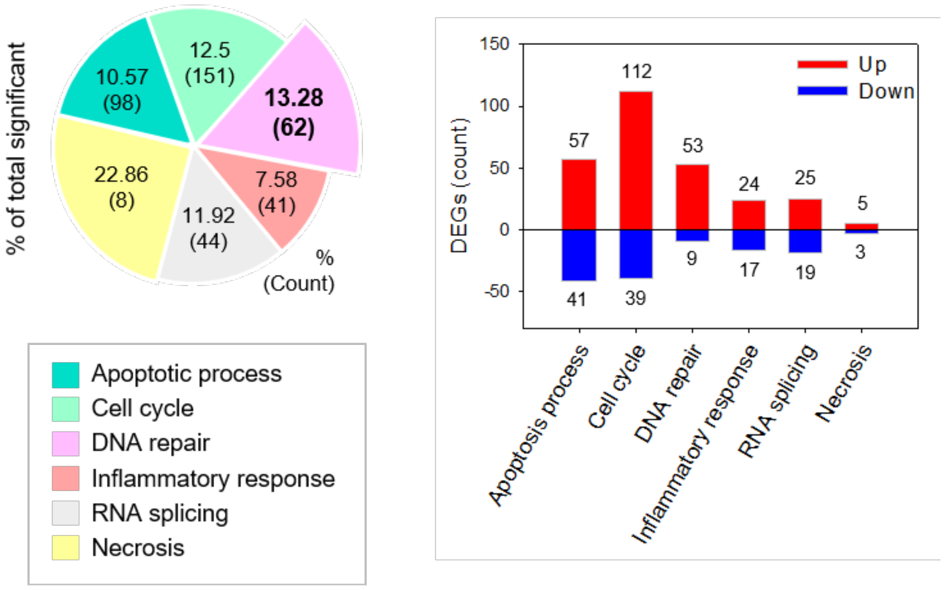

Figure S5. Analysis of differences in gene ontology between GuaRD-- and PEI-treated cells.

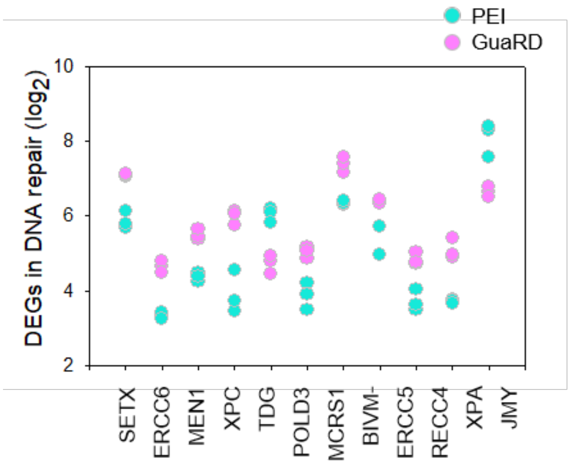

Figure S6. Gene expression graph of the top 12 significant genes in DNA repair gene set.

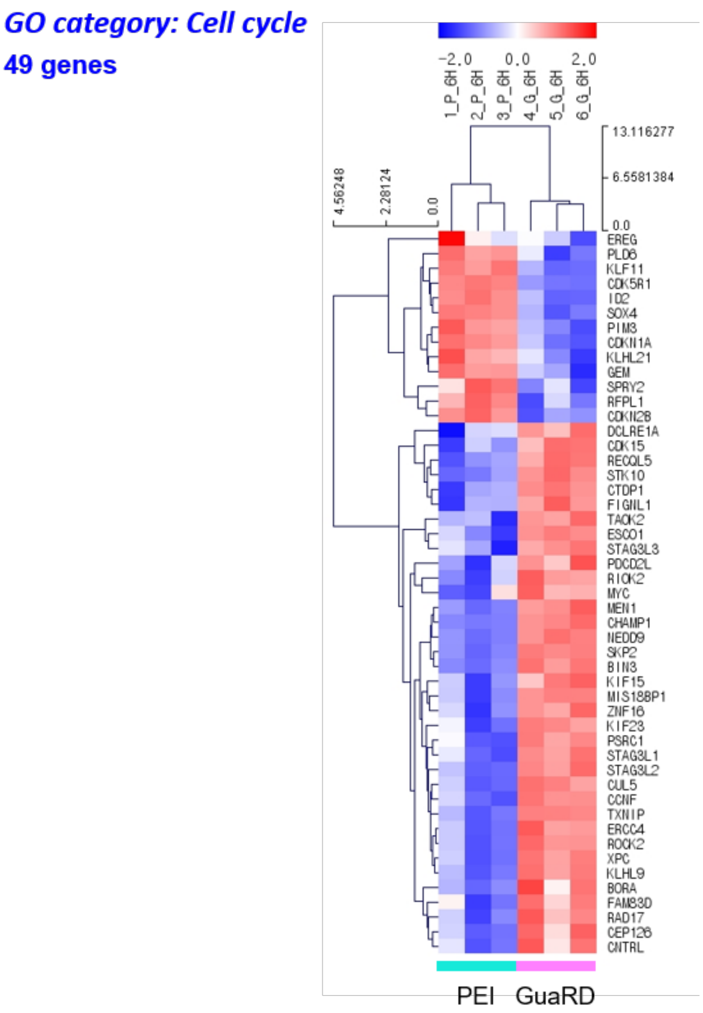

Figure S7. Heat map of the cell cycle gene set.

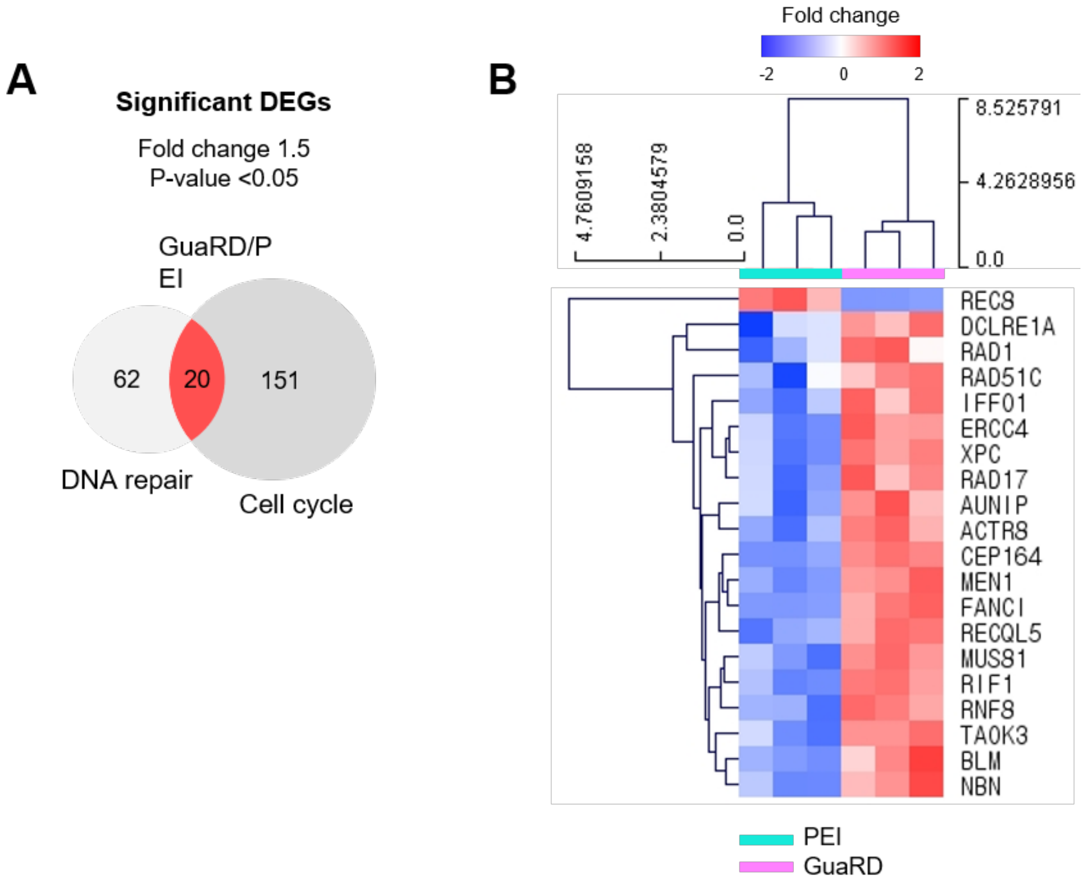

Figure S8. Significant differential expression genes (DEGs) between DNA repair and the cell cycle.

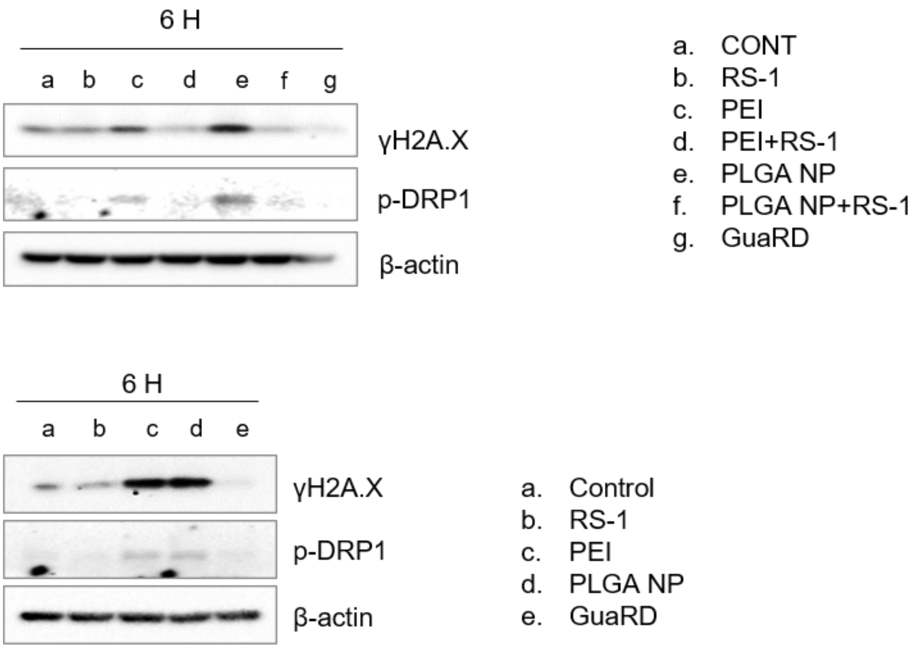

Figure S9. Evaluation of genomic and mitochondrial damage by western blotting.

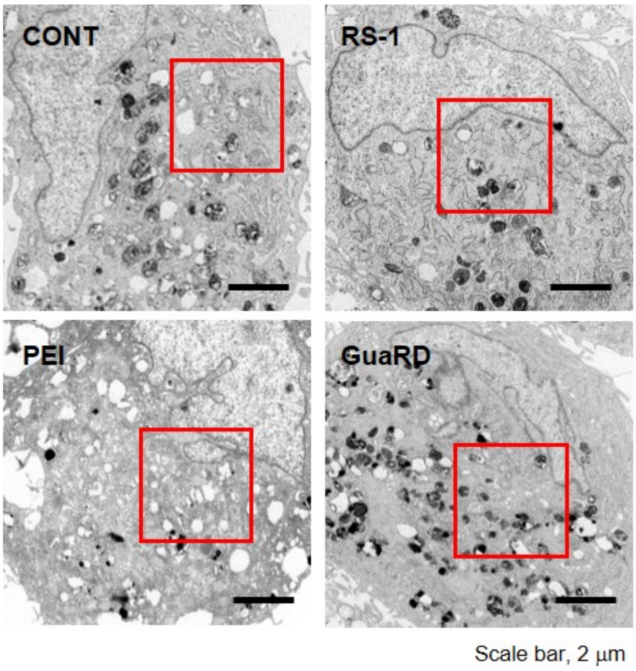

Figure S10. Mitochondrial structure images acquired using TEM.

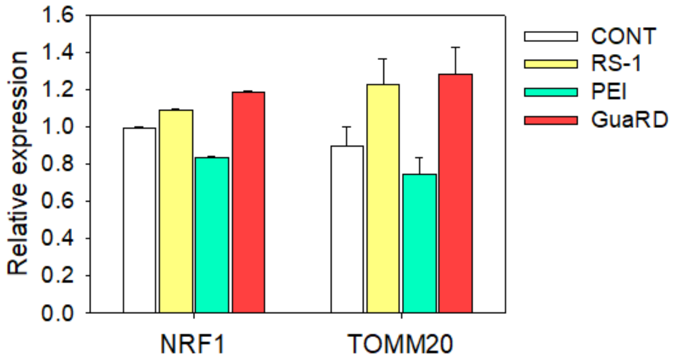

Figure S11. Quantitative graph for Figure 5F.

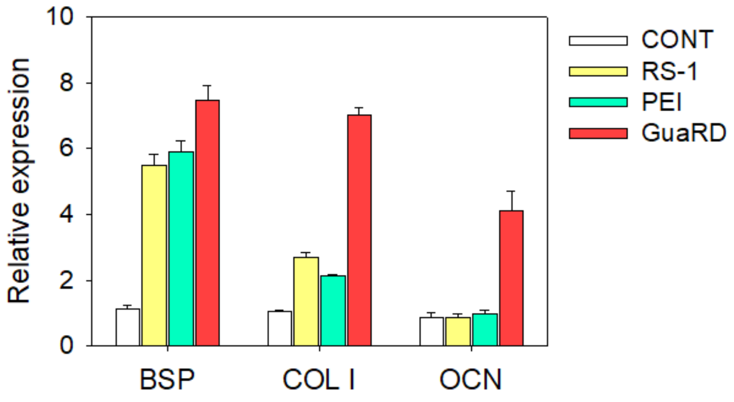

Figure S12. Quantitative graph for Figure 6B.

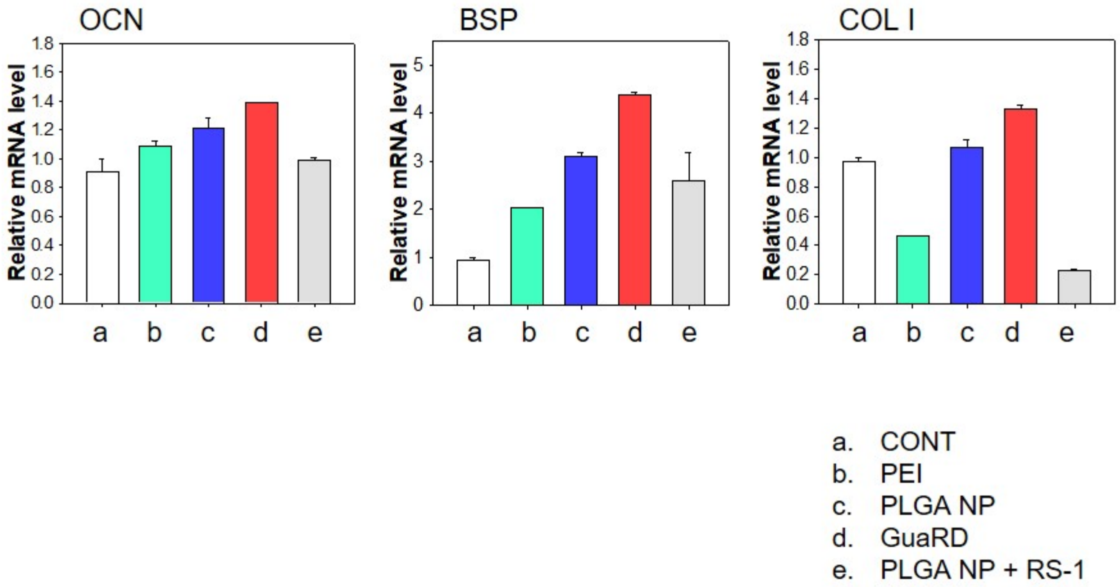

Figure S13. Evaluation of osteogenic marker expression in the 3D culture system.

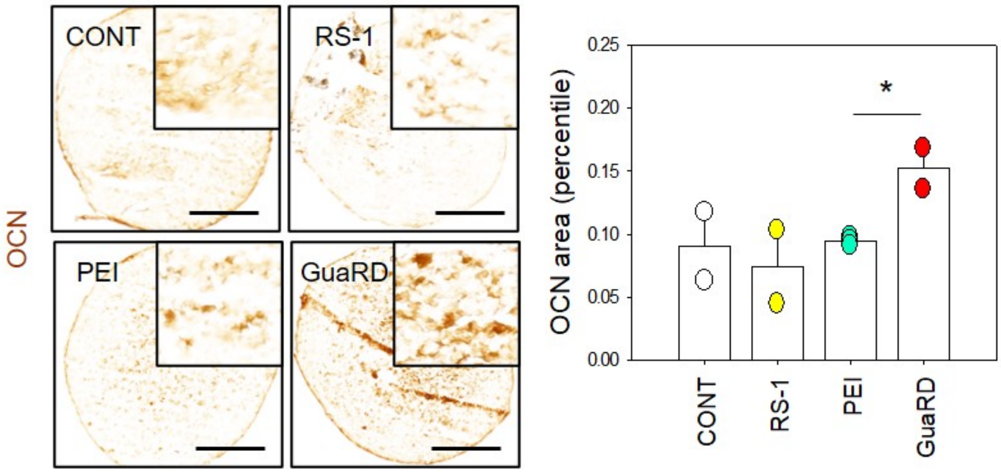

Figure S14. OCN expression analyzed by DAB staining.
